# Ethyl 4-ethyl­amino-3-nitro­benzoate

**DOI:** 10.1107/S1600536808043602

**Published:** 2009-01-08

**Authors:** Hao-Yuan Li, Bo-Nian Liu, Shi-Gui Tang, Cheng Guo

**Affiliations:** a College of Life Science and Pharmaceutical Engineering, Nanjing University of Technology, Nanjing 210009, People’s Republic of China; b College of Science, Nanjing University of Technolgy, Xinmofan Road No.5 Nanjing, Nanjing 210009, People’s Republic of China

## Abstract

In the mol­ecule of the title compound, C_11_H_14_N_2_O_4_, a bifurcated intra/intermolecular N—H⋯(O,O) hydrogen bond occurs.The intramolecular component results in a non-planar six-membered ring with a flattened-boat conformation. In the crystal structure, the inter­molecular interaction links the mol­ecules into chains parallel to the *b* axis.

## Related literature

For a related structure, see: Ates-Alagoz *et al.* (2001 ▶). For bond-length data, see: Allen *et al.* (1987 ▶). For ring-puckering parameters, see: Cremer & Pople (1975 ▶).
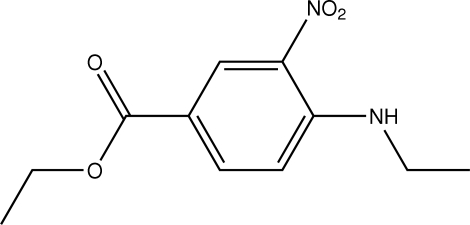


## Experimental

### 

#### Crystal data

C_11_H_14_N_2_O_4_
*M* *_r_* = 238.24
Monoclinic, 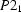

*a* = 4.2360 (8) Å
*b* = 16.180 (3) Å
*c* = 8.4890 (17) Å
β = 95.80 (3)°
*V* = 578.8 (2) Å^3^
*Z* = 2
Mo *K*α radiation
μ = 0.11 mm^−1^
*T* = 294 (2) K
0.30 × 0.20 × 0.10 mm

#### Data collection

Enraf–Nonius CAD-4 diffractometer
Absorption correction: ψ scan (North *et al.*, 1968 ▶) *T*_min_ = 0.969, *T*_max_ = 0.990
1213 measured reflections
1066 independent reflections
841 reflections with *I* > 2σ(*I*)
*R*_int_ = 0.018
3 standard reflections frequency: 120 min intensity decay: none

#### Refinement

*R*[*F* ^2^ > 2σ(*F* ^2^)] = 0.077
*wR*(*F* ^2^) = 0.173
*S* = 1.01
1066 reflections
154 parameters
4 restraints
H-atom parameters constrained
Δρ_max_ = 0.24 e Å^−3^
Δρ_min_ = −0.30 e Å^−3^

### 

Data collection: *CAD-4 Software* (Enraf–Nonius, 1989 ▶); cell refinement: *CAD-4 Software*; data reduction: *XCAD4* (Harms & Wocadlo, 1995 ▶); program(s) used to solve structure: *SHELXS97* (Sheldrick, 2008 ▶); program(s) used to refine structure: *SHELXL97* (Sheldrick, 2008 ▶); molecular graphics: *ORTEP-3 for Windows* (Farrugia, 1997 ▶); software used to prepare material for publication: *SHELXL97*.

## Supplementary Material

Crystal structure: contains datablocks global, I. DOI: 10.1107/S1600536808043602/hk2598sup1.cif

Structure factors: contains datablocks I. DOI: 10.1107/S1600536808043602/hk2598Isup2.hkl

Additional supplementary materials:  crystallographic information; 3D view; checkCIF report

## Figures and Tables

**Table 1:** Hydrogen-bond geometry (Å, °)

*D*—H⋯*A*	*D*—H	H⋯*A*	*D*⋯*A*	*D*—H⋯*A*
N1—H1*A*⋯O2	0.86	2.00	2.645 (10)	131
N1—H1*A*⋯O3^i^	0.86	2.45	3.053 (10)	128

Symmetry code: (i) 
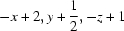
.

**Fig. 1.**
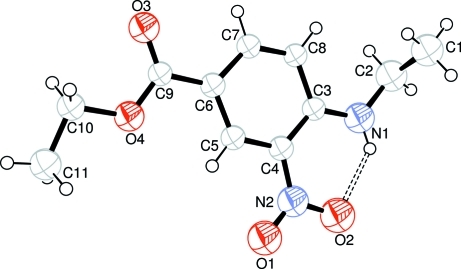
The molecular structure of the title molecule, with the atom-numbering scheme. Hydrogen bond is shown as dashed line.

**Fig. 2.**
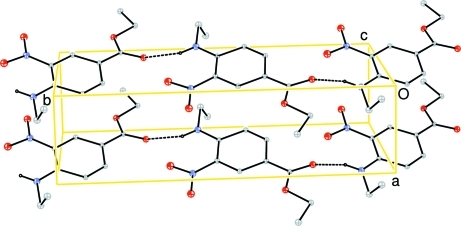
A partial packing diagram of the title compound. Hydrogen bonds are shown as dashed lines.

## supplementary crystallographic information

### Comment

Some derivatives of benzoic acid are important chemical materials. We report herein the crystal structure of the title compound.

In the molecule of the title compound (Fig 1), the bond lengths (Allen *et al.*,  1987) and angles are within normal ranges. Ring A (C3-C8) is, of course, planar. The intramolecular N-H···O hydrogen bond (Table 1) results in a nonplanar six-membered ring B (O2/N1/N2/C3/C4/H1A), having total puckering amplitude, Q_T_, of 0.163 (2) Å, flattened-boat conformation [φ = 52.00 (3)° and θ = 19.29 (4)°] (Cremer & Pople, 1975).

In the crystal structure, intermolecular N-H···O hydrogen bonds (Table 1) link the molecules into chains parallel to the b axis (Fig. 2), in which they may be effective in the stabilization of the structure.

### Experimental

For the preparation of the title compound, ethyl 4-chloro-3-nitrobenzoate (5.3 g, 0.023 mol) was refluxed in ethyl amine (20 ml) and tetrahydrofuran (50 ml) for 2 h. Then, solvents were evaporated and water was added to give yellow precipate. It was collected by filtration and washed with cold ethanol (2 X 15 ml) to afford the yellow solid (yield; 4.4 g, 80%) (Ates-Alagoz *et al.*, 2001). Crystals suitable for X-ray analysis were obtained by slow evaporation of an ethanol solution.

### Refinement

H atoms were positioned geometrically, with N-H = 0.86 Å (for NH) and C-H = 0.93, 0.97 and 0.96 Å for aromatic, methylene and methyl H, respectively, and constrained to ride on their parent atoms, with U_iso_(H) = xU_eq_(C,N), where x = 1.5 for methyl H and x = 1.2 for all other H atoms.

### Crystal data

**Table:**

C_11_H_14_N_2_O_4_	*F*(000) = 252
*M**_r_* = 238.24	*D*_x_ = 1.367 Mg m^−^^3^
Monoclinic, *P*2_1_	Mo *K*α radiation, λ = 0.71073 Å
Hall symbol: P 2yb	Cell parameters from 25 reflections
*a* = 4.2360 (8) Å	θ = 10–12°
*b* = 16.180 (3) Å	µ = 0.11 mm^−^^1^
*c* = 8.4890 (17) Å	*T* = 294 K
β = 95.80 (3)°	Block, colorless
*V* = 578.8 (2) Å^3^	0.30 × 0.20 × 0.10 mm
*Z* = 2	

### Data collection

**Table:**

Enraf–Nonius CAD-4 diffractometer	841 reflections with *I* > 2σ(*I*)
Radiation source: fine-focus sealed tube	*R*_int_ = 0.018
graphite	θ_max_ = 25.2°, θ_min_ = 2.4°
ω/2θ scans	*h* = −5→5
Absorption correction: ψ scan (North *et al.*, 1968)	*k* = 0→19
*T*_min_ = 0.969, *T*_max_ = 0.990	*l* = 0→10
1213 measured reflections	3 standard reflections every 120 min
1066 independent reflections	intensity decay: none

### Refinement

**Table:**

Refinement on *F*^2^	Primary atom site location: structure-invariant direct methods
Least-squares matrix: full	Secondary atom site location: difference Fourier map
*R*[*F*^2^ > 2σ(*F*^2^)] = 0.077	Hydrogen site location: inferred from neighbouring sites
*wR*(*F*^2^) = 0.173	H-atom parameters constrained
*S* = 1.01	*w* = 1/[σ^2^(*F*_o_^2^) + (0.05*P*)^2^ + 1.25*P*] where *P* = (*F*_o_^2^ + 2*F*_c_^2^)/3
1066 reflections	(Δ/σ)_max_ < 0.001
154 parameters	Δρ_max_ = 0.24 e Å^−^^3^
4 restraints	Δρ_min_ = −0.30 e Å^−^^3^

### Special details

**Table:**

Geometry. All e.s.d.'s (except the e.s.d. in the dihedral angle between two l.s. planes) are estimated using the full covariance matrix. The cell e.s.d.'s are taken into account individually in the estimation of e.s.d.'s in distances, angles and torsion angles; correlations between e.s.d.'s in cell parameters are only used when they are defined by crystal symmetry. An approximate (isotropic) treatment of cell e.s.d.'s is used for estimating e.s.d.'s involving l.s. planes.
Refinement. Refinement of *F*^2^ against ALL reflections. The weighted *R*-factor *wR* and goodness of fit *S* are based on *F*^2^, conventional *R*-factors *R* are based on *F*, with *F* set to zero for negative *F*^2^. The threshold expression of *F*^2^ > σ(*F*^2^) is used only for calculating *R*-factors(gt) *etc*. and is not relevant to the choice of reflections for refinement. *R*-factors based on *F*^2^ are statistically about twice as large as those based on *F*, and *R*- factors based on ALL data will be even larger.

### Fractional atomic coordinates and isotropic or equivalent isotropic displacement parameters (Å^2^)

**Table:**

	*x*	*y*	*z*	*U*_iso_*/*U*_eq_	
O1	0.7012 (15)	1.1050 (4)	0.7940 (7)	0.0772 (18)	
O2	0.9134 (16)	1.1622 (4)	0.5988 (8)	0.083 (2)	
O3	0.9764 (13)	0.7330 (4)	0.7615 (6)	0.0589 (15)	
O4	0.7965 (13)	0.8254 (3)	0.9244 (6)	0.0564 (14)	
N1	1.2210 (17)	1.0712 (5)	0.4054 (8)	0.065 (2)	
H1A	1.1591	1.1204	0.4256	0.078*	
N2	0.8667 (17)	1.0957 (5)	0.6810 (9)	0.0659 (19)	
C1	1.212 (2)	1.0329 (6)	0.1174 (10)	0.071 (2)	
H1B	1.3443	1.0259	0.0328	0.106*	
H1C	1.0571	1.0750	0.0891	0.106*	
H1D	1.1069	0.9818	0.1355	0.106*	
C2	1.414 (2)	1.0581 (6)	0.2654 (10)	0.071 (3)	
H2A	1.5246	1.1089	0.2453	0.085*	
H2B	1.5719	1.0157	0.2925	0.085*	
C3	1.1440 (15)	1.0085 (4)	0.4988 (7)	0.0383 (15)	
C4	0.9754 (15)	1.0194 (5)	0.6315 (8)	0.0416 (16)	
C5	0.9015 (15)	0.9543 (4)	0.7232 (8)	0.0401 (17)	
H5A	0.7858	0.9646	0.8086	0.048*	
C6	0.9927 (16)	0.8715 (4)	0.6946 (8)	0.0404 (16)	
C7	1.1639 (14)	0.8632 (4)	0.5582 (7)	0.0393 (16)	
H7A	1.2333	0.8105	0.5348	0.047*	
C8	1.2314 (16)	0.9232 (4)	0.4633 (8)	0.0377 (16)	
H8A	1.3342	0.9118	0.3740	0.045*	
C9	0.9235 (16)	0.8047 (4)	0.7942 (8)	0.0376 (15)	
C10	0.7087 (18)	0.7563 (5)	1.0314 (8)	0.0483 (19)	
H10A	0.8916	0.7222	1.0650	0.058*	
H10B	0.5432	0.7217	0.9787	0.058*	
C11	0.590 (2)	0.8020 (6)	1.1726 (9)	0.060 (2)	
H11A	0.5211	0.7625	1.2463	0.090*	
H11B	0.4157	0.8372	1.1357	0.090*	
H11C	0.7590	0.8348	1.2240	0.090*	

### Atomic displacement parameters (Å^2^)

**Table:**

	*U*^11^	*U*^22^	*U*^33^	*U*^12^	*U*^13^	*U*^23^
O1	0.095 (4)	0.068 (4)	0.073 (4)	0.010 (4)	0.028 (4)	0.005 (4)
O2	0.094 (5)	0.079 (5)	0.078 (5)	−0.004 (4)	0.019 (4)	0.003 (4)
O3	0.068 (3)	0.052 (4)	0.059 (3)	0.004 (3)	0.015 (3)	0.002 (3)
O4	0.070 (3)	0.049 (3)	0.051 (3)	0.005 (3)	0.009 (3)	−0.002 (3)
N1	0.072 (4)	0.063 (5)	0.059 (4)	−0.011 (4)	0.005 (4)	−0.008 (4)
N2	0.068 (4)	0.068 (5)	0.061 (4)	0.001 (4)	0.002 (4)	−0.003 (4)
C1	0.083 (6)	0.070 (6)	0.061 (5)	0.008 (5)	0.012 (4)	0.004 (5)
C2	0.071 (5)	0.075 (7)	0.067 (6)	0.008 (5)	0.012 (4)	−0.005 (5)
C3	0.043 (3)	0.037 (4)	0.033 (3)	−0.006 (3)	−0.003 (3)	0.003 (3)
C4	0.040 (3)	0.045 (4)	0.039 (4)	−0.002 (3)	0.003 (3)	0.004 (3)
C5	0.039 (3)	0.043 (4)	0.038 (4)	0.001 (3)	0.002 (3)	−0.001 (3)
C6	0.044 (4)	0.034 (4)	0.043 (4)	0.002 (3)	0.006 (3)	−0.002 (3)
C7	0.044 (4)	0.037 (4)	0.037 (4)	0.004 (3)	0.005 (3)	−0.002 (3)
C8	0.050 (4)	0.029 (4)	0.035 (4)	−0.005 (3)	0.009 (3)	0.005 (3)
C9	0.047 (4)	0.029 (4)	0.037 (4)	0.002 (3)	0.004 (3)	−0.001 (3)
C10	0.055 (4)	0.052 (5)	0.038 (4)	0.002 (4)	0.005 (3)	0.006 (4)
C11	0.062 (5)	0.064 (5)	0.053 (5)	0.003 (4)	0.007 (4)	−0.002 (4)

### Geometric parameters (Å, °)

**Table:**

O1—N2	1.253 (9)	C3—C8	1.467 (9)
O2—N2	1.308 (10)	C4—C5	1.365 (10)
O3—C9	1.219 (9)	C5—C6	1.422 (9)
O4—C9	1.320 (8)	C5—H5A	0.9300
O4—C10	1.510 (8)	C6—C9	1.420 (9)
N1—C2	1.523 (11)	C6—C7	1.434 (9)
N1—C3	1.349 (10)	C7—C8	1.311 (9)
N1—H1A	0.8600	C7—H7A	0.9300
N2—C4	1.397 (10)	C8—H8A	0.9300
C1—C2	1.502 (12)	C10—C11	1.535 (10)
C1—H1B	0.9600	C10—H10A	0.9700
C1—H1C	0.9600	C10—H10B	0.9700
C1—H1D	0.9600	C11—H11A	0.9600
C2—H2A	0.9700	C11—H11B	0.9600
C2—H2B	0.9700	C11—H11C	0.9600
C3—C4	1.405 (9)		
			
C2—N1—H1A	118.9	C4—C5—H5A	118.4
C3—N1—C2	122.3 (8)	C6—C5—H5A	118.4
C3—N1—H1A	118.9	C9—C6—C5	122.6 (6)
O1—N2—O2	115.9 (8)	C9—C6—C7	124.2 (6)
O1—N2—C4	124.3 (8)	C5—C6—C7	113.2 (6)
O2—N2—C4	119.6 (7)	C8—C7—C6	126.0 (7)
C9—O4—C10	117.5 (6)	C8—C7—H7A	117.0
C2—C1—H1B	109.5	C6—C7—H7A	117.0
C2—C1—H1C	109.5	C7—C8—C3	119.6 (6)
H1B—C1—H1C	109.5	C7—C8—H8A	120.2
C2—C1—H1D	109.5	C3—C8—H8A	120.2
H1B—C1—H1D	109.5	O3—C9—O4	122.1 (7)
H1C—C1—H1D	109.5	O3—C9—C6	122.3 (6)
C1—C2—N1	112.7 (7)	O4—C9—C6	115.6 (6)
C1—C2—H2A	109.0	O4—C10—C11	103.4 (6)
N1—C2—H2A	109.0	O4—C10—H10A	111.1
C1—C2—H2B	109.0	C11—C10—H10A	111.1
N1—C2—H2B	109.0	O4—C10—H10B	111.1
H2A—C2—H2B	107.8	C11—C10—H10B	111.1
N1—C3—C4	123.3 (7)	H10A—C10—H10B	109.0
N1—C3—C8	120.4 (6)	C10—C11—H11A	109.5
C4—C3—C8	116.3 (6)	C10—C11—H11B	109.5
C5—C4—N2	114.2 (6)	H11A—C11—H11B	109.5
C5—C4—C3	121.6 (7)	C10—C11—H11C	109.5
N2—C4—C3	124.2 (7)	H11A—C11—H11C	109.5
C4—C5—C6	123.2 (6)	H11B—C11—H11C	109.5
			
C3—N1—C2—C1	84.9 (10)	C4—C5—C6—C7	−1.2 (9)
C2—N1—C3—C4	177.9 (6)	C9—C6—C7—C8	179.8 (7)
C2—N1—C3—C8	−3.1 (11)	C5—C6—C7—C8	−1.2 (9)
O1—N2—C4—C5	−3.6 (10)	C6—C7—C8—C3	3.4 (10)
O2—N2—C4—C5	−177.5 (7)	N1—C3—C8—C7	177.8 (7)
O1—N2—C4—C3	175.8 (7)	C4—C3—C8—C7	−3.1 (9)
O2—N2—C4—C3	1.9 (10)	C10—O4—C9—O3	−1.7 (10)
N1—C3—C4—C5	179.9 (7)	C10—O4—C9—C6	178.0 (6)
C8—C3—C4—C5	0.9 (8)	C5—C6—C9—O3	173.2 (7)
N1—C3—C4—N2	0.6 (10)	C7—C6—C9—O3	−7.9 (11)
C8—C3—C4—N2	−178.5 (6)	C5—C6—C9—O4	−6.5 (10)
N2—C4—C5—C6	−179.3 (6)	C7—C6—C9—O4	172.4 (6)
C3—C4—C5—C6	1.3 (9)	C9—O4—C10—C11	176.7 (6)
C4—C5—C6—C9	177.8 (6)		

### Hydrogen-bond geometry (Å, °)

**Table:**

*D*—H···*A*	*D*—H	H···*A*	*D*···*A*	*D*—H···*A*
N1—H1A···O2	0.86	2.00	2.645 (10)	131
N1—H1A···O3^i^	0.86	2.45	3.053 (10)	128

Symmetry codes: (i) −*x*+2, *y*+1/2, −*z*+1.
